# Modified creatine greatly increases the performance of skeletal and smooth muscles

**DOI:** 10.1016/j.bbrep.2025.101934

**Published:** 2025-01-28

**Authors:** Anatoly Soloviev, Vadym Kozlovsky, Dmytro Nozdrenko, Vadym Sydorenko, Igor Monchak, Natalia Vdovenko, Olena Maidaniuk, Volodymyr Fetyukhin

**Affiliations:** a Department for Pharmacology of Cellular Signal Systems and Experimental Therapeutics, Institute of Pharmacology and Toxicology, National Academy of Medical Sciences of Ukraine, Kyiv, 03057, Ukraine; b LLC “MEDIVALEX”, Prague, 15200, Czech Republic; c ESC “Institute of Biology and Medicine”, Taras Shevchenko National University of Kyiv, 03127, Ukraine; d Department of Medical and Biological Disciplines, State Scientific Research Institute of Physical Culture and Sports, 03131, Kyiv, Ukraine; e I.F. Lab, LLC, Life Chemicals Inc. Distributor, Kyiv, 02094, Ukraine

**Keywords:** Creatine, Creatine phosphokinase, Fatigue, Lactate, Skeletal muscle, Portal vein

## Abstract

Creatine is a nitrogen-containing carboxylic acid and a main component of phosphocreatine. In recent years, creatine is considered as a component of dietary nutrition, to improve the efficiency of physical activity and increase muscle mass of athletes and older people. Creatine has been shown to be able restore cardiac contractility impairment after myocardial infarction. However, as muscle cells do not synthesise creatine, the efficiency of creatine depends on its transmembrane transport.

In our study, we evaluated the effect of «ProCreatine» (ProCr), a novel membrane transporter-independent creatine modification on fatigability of the rat gastrocnemius muscle and portal vein smooth muscle using fatigue stimulation pools. Mechanokinetic and biomechanical markers of fatigue in muscles to maintain the level of isometric tension induced by field electrical stimulation were examined. The results indicate that administration of ProCr to skeletal muscle significantly increases maximal force output, integrated muscle contractile force and significantly increases muscle productivity. We observed positive changes in all studied biochemical indices of fatigue. In addition, ProCr increases the duration of sustaining a constant level of isometric contraction in portal vein smooth muscle caused by electrical stimulation by 6 fold. Regular creatine in the same dose had no significant effect on these parameters neither in skeletal nor in smooth muscles. The data obtained suggest the possibility of using ProCr as a therapeutic agent capable of reducing and correcting pathological conditions of the muscular system that arise during the processes of fatigue in skeletal muscles and smooth muscles of hollow organs.

## Highlights

• New creatine restored the performance and biochemical markers of fatigue in the rat gastrocnemius and portal vein muscles.

## 1 Introduction

Muscle fatigue is the main and natural protective mechanism against overexertion of skeletal muscles, preventing the development of undesirable processes in muscle tissue [1]. This process involves numerous functional systems: from the central nervous system (CNS) to the contractile apparatus of muscles. To date, there is no single global mechanism responsible for the development of muscle fatigue. Factors such as the process of converting adenosine triphosphate (ATP) into adenosine diphosphate (ADP), a decrease in pH levels, and disruption of myosin phosphorylation processes, even when considered together, cannot fully explain the inhibition of actomyosin interaction during the development of fatigue [2]. The development of muscle fatigue is usually considered as a decrease in the maximum force developed, while submaximal contractions can persist even after the development of muscle fatigue [3].

Creatine is a well-known nitrogen-containing carboxylic acid that is consumed both exogenously with food and synthesized endogenously through a complex interorgan process. It functions in conjunction with creatine kinase, and it is creatine and its phosphorylated form, phosphocreatine, that play a decisive role in maintaining ATP concentrations in tissues with high energy requirements, such as skeletal muscle, heart, and brain. Creatine is synthesized from the amino acids glycine, arginine, and methionine by the enzymes glycine amidinotransferase, guanidine acetate methyltransferase, and methionine adenosyltransferase, and is stored in cells as phosphocreatine [4].

In addition to direct participation in the regeneration of ATP molecules, phosphocreatine has the ability to neutralize acids that are formed during muscle activity and reduces the blood pH level, thereby preventing the development of muscle fatigue. It is also known that creatine can activate glycolysis, which helps maintain muscle performance in conditions of oxygen deficiency [5]. In heart failure, creatine and phosphocreatine levels also reduced due to decreased expression of the creatine transporter. This leads to a decrease in the reserve of myocardial contractility and is extremely dangerous for the patient. Creatine is successfully used in the treatment of patients with cardiovascular pathology, increasing the efficiency of the heart muscle in conditions of already developed heart failure [6].

Although creatine supplementation appears to have significant therapeutic potential, the scientific literature that confirms a role of creatine in vascular health remains limited. A review by Clarke et al. [7] demonstrated the ability of creatine to increase natural stores of high-energy metabolites in endothelial cells and its ability to neutralize free radicals. It is able to protect DNA and RNA from cytotoxic stimuli and to increase the efficiency of endothelial nitric oxide synthase (eNOS) and, accordingly, nitric oxide (NO) synthesis and bioavailability. Creatine also significantly increases microvascular density and vasomotor function, enhances endothelial cell membrane stability, and is able to improve energy-dependent ion pumps that support endothelial and smooth muscle function.

The effectiveness of various other forms of creatine other than the classic form is questionable due to a lack of real scientific data, rather than extrapolation of data from manufacturers of alternative [8,9]. To date, there is no consensus in the methodological assessment of the effect of creatine and its analogues on the efficiency of muscular system itself.

We hypothesized that a possible main problem that limits the effectiveness of creatine may be its direct transport into the cells, performed through a specific sodium-dependent transporter SLC6A8. Thus, SLC6A8 plays an important role in muscle physiology, and inhibition of creatine transport in experimental animals causes muscle weakness [10]. Upregulation of the creatine transporter and the resulting increase in intracellular creatine concentration should be considered as a potential neuro- or cardioprotective mechanism [11]. In case of impaired transmembrane transport, forms of creatine capable of passive diffusion across the membrane may have advantages. For example, cyclocreatine, a cyclic analogue of creatine, freely penetrates membranes and is effectively phosphorylated and dephosphorylated by phosphokinase, increasing ATP levels and having a protective effect [12].

We used a new molecular construct of creatine monohydrate, which has a potent ergogenic effect (laboratory name « ProCreatine» (ProCr). The unique properties of ProCr are attributed to the modification of its structure, which provided for inclusion in its molecule of a compound that is equally soluble in water, alcohol and non-polar solvents. This allowed for its direct penetration through cell membranes, without the involvement of a membrane transporter, as the effectiveness of standard creatine monohydrate is limited by its carriers. Standard creatine monohydrate administration schemes involve a so-called “loading” phase, and the need to use sufficiently large doses to achieve an effect. The purpose of developing ProCreatine was precisely the need to achieve an effect without the “loading” phase, which would ensure its direct effect on the cell, in particular, on cellular energy metabolism.

The main goal of this study was to investigate and compare the effect of creatine monohydrate and ProCr in vivo (skeletal muscles) and in vitro (vascular smooth muscles) on performance and development of fatigue processes in the muscular system with defined and controlled parameters of the tested models.

## 2 Materials and methods

### 2.1 Animals

All animal studies, including skeletal and smooth muscles were performed in strict accordance with the recommendations of the «European Convention for the Protection of Vertebrate Animals used for Experimental and other Scientific Purposes» and approved by the Institutional Animal Care and Use Committees in accordance with the Law of Ukraine No. 3446 - IV 02/21/2006, Kyiv, “On the Protection of Animals from Cruelty when conducting biomedical research”. Experiments were performed on 1–6 weeks male Wistar rats (weight 170–300 g) housed under controlled environmental conditions (21 °C, 12 h–12 h light–dark cycle) and free access to water and standard rodent chow.

### 2.2 Physicochemical properties of ProCreatine

To confirm the potentially higher lipophilic character of ProCreatin compared to standard creatine monohydrate, a series of chemical studies performed to test its solubility and lipophilicity by analyzing its distribution in the octanol-water system. The solubility of creatine monohydrate was established to be 21 mg/mL, which is generally consistent with the literature data [13], where the solubility of creatine monohydrate was established at 17 mg/mL. A slight difference from our data can be explained by different manufacturers and the degree of purification and particle size of the substance. The solubility of ProCreatine (200 Mesh) was 755 mg/ml, which is almost 50 times greater.

In the paper cited above, relatively low octanol-water partition coefficients (log P from −3.8 to −3.2) were obtained for each creatine salt or zwitterionic monohydrate, which indicate a very low affinity to the lipophilic phase of octanol. By contrast, for ProCreatine this value was +0.97 log P at 25 °C, indicating the presence of pronounced lipophilic properties (data not shown).

### 2.3 Biomechanical analysis of skeletal muscle contractile activity

Muscle fatigue was induced by successive pulses of electrical stimulation with a frequency of 1 Hz for 1600 and with a frequency of 50 Hz for 5 s without a relaxation period between them. Anaesthesia was performed by intraperitoneal injection of Nembutal (40 mg/kg). Standard preparation for the experiment included cannulation (a. carotis communis sinistra) for measuring arterial pressure and laminectomy at the level of the lumbar spinal cord of the rat. The gastrocnemius muscle was freed from surrounding tissues, and the distal part of its tendon was connected to a force measuring transducer. To prepare for modulated stimulation of efferents in the corresponding segments of the rat spinal cord, the ventral roots were cut immediately at their exit from the spinal cord. The dynamic properties of muscle contraction were studied under conditions of muscle activation by the method of modulated stimulation as described previously [14]. External load on the muscle was controlled using a system of mechanical stimulators. Using strain gauges, the integral power (calculated area under the force curve) was measured, which is an indicator of the overall work of the muscle under the conditions of the applied stimulation parameters [15].

Test substances creatine monohydrate and ProCr were dissolved in saline solution and administered at a single dose of 1 mg/kg in three injections, equally spaced from each other. In this study, the administration was performed specifically in the gastrocnemius muscle using a TTFK-1.2230 microdoser. This process was visually monitored using a Grandway FIM-17 fiber optic microscope. Usually, maintaining increased creatine levels in muscles is achieved by taking the supplement in a dose of 2–3 g daily. Thus, the dosages we use are at least 20 times lower than those recommended for practical use.

### 2.4 Experiments in vascular smooth muscles

Male Wistar rats (weight 250–275 g) were anesthetized with urethane (400 mg/kg v/v, intravenously) and alpha-chloralose (40 mg/kg), and then euthanized by dislocation of the cervical spines. After the euthanization procedure, the rat portal vein segment was dissected and carefully cleared of both connective and adipose tissues in modified Krebs buffer of the following constitution (mM): NaCl, 133; KC1, 4.7; NaHCO_3_, 10; NaH_2_PO_4_, 1.38; CaC1_2_, 2.5; MgCl_2_, 1.2; HEPES, 10; glucose, 7.8; pH, 7.3 at temperature 37 °C. Then, portal vein was cut into longitudinal strips 1 mm wide and 3–4 mm long and mounted in a 0.6 mL tissue bath between a fixed stainless steel hook and an isometric force transducer (AE 801, SensoNor, A/S, Norten, Norway) connected to an analog-to-digital converter (ADC LabTrax-4/16 (World Precision Instruments, Inc., Sarasota, USA).

The organ tissue bath was equipped with platinum-Ag/Cl polarographic electrodes connected with polarographic analyzer PA-3 (Laboratorni Pristroje, Praha, Czech Republic) and stimulating platinum electrodes (electronic stimulator SEN-1101, Nihon Cohden, Tokyo, Japan). The vascular strips were lengthened sufficiently to produce a passive force in a range of 4–6 mN. Then the strips were equilibrated for 1 h at a resting tension until a stable level of isometric tension is achieved. Following the equilibration period, the aortic rings were exposed several times to norepinephrine (NE, 10^−6^ M) or electrical simulation (13 V, 20 msec square pulses, 8 Hz) until reproducible contractile responses were obtained. Test substance ProCr was applied in tissue bath at a concentration of 10^−4^ M. The data obtained during the experiment were recorded using an ADC LabTrax4/16 and a LabScribe2 software (iWorx Inc, NH, USA). The saved data were filtered and processed in LabScribe2.

### 2.5 Chemicals

All compounds were obtained from Sigma Aldrich (St. Louis, USA). ProCreatine was obtained from I·F.Lab (Ukraine).

### 2.6 Biochemical analyzes

The level of biochemical markers of muscle fatigue in the blood of experimental animals (creatinine, creatine phosphokinase, lactate, lactate dehydrogenase) was determined using a biochemical analyzer JN-1101-TR2 (Netherlands).

### 2.7 Statistics and analysis

Statistical analysis was performed by methods of variation statistics using Origin 9.3 software (OriginLab Corporation, USA). At least six repetitions were performed for each measurement. Data are expressed as means ± SEM for each group and *n* indicates the number of preparations or animals tested. Differences between experimental groups were determined using one-way ANOVA followed by Bonferroni's multiple comparison test. Differences at p < 0.05 were considered to be significant.

## 3 Results

### 3.1 ProCr restores skeletal muscle functionality during fatigue

**Fig. 1:**
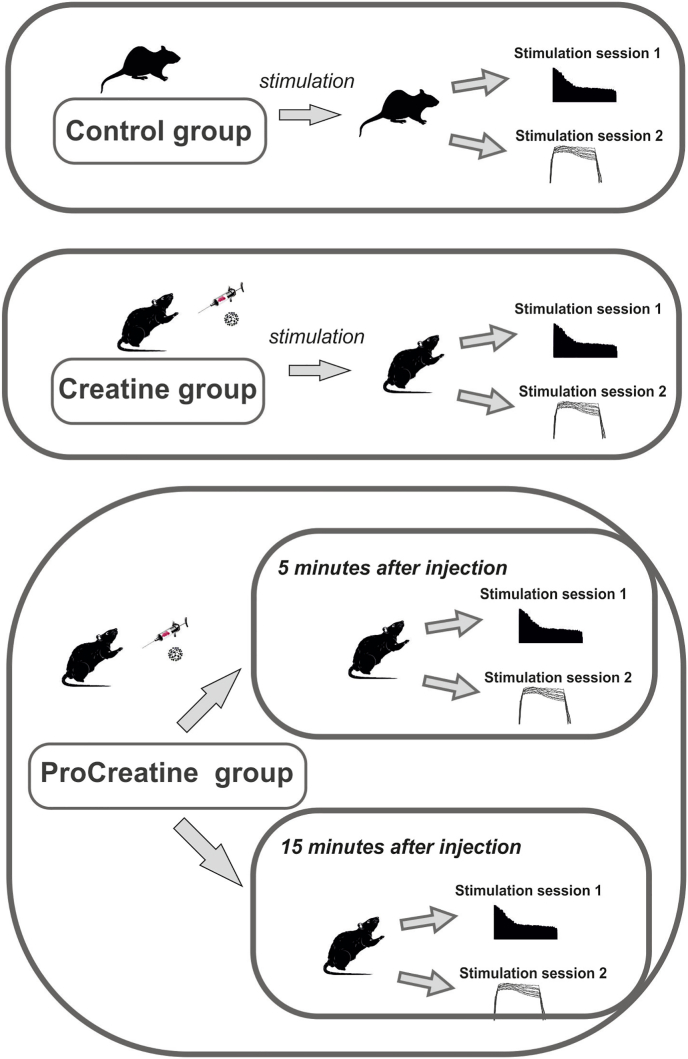
Experimental scheme to study the effect of modified creatine on fatigue in rat skeletal muscles. The animals were divided into three groups in which they received muscle stimulation in two sessions: long-term stimulation with a 1 Hz frequency and tetanic stimulation with a 50 Hz frequency and a 5 s duration. The « Creatine » group was injected with regular creatine (1 mg/kg b. w., single dose), the «ProCreatine » group was injected with a modified form of creatine (same protocol).

The scheme of the experiment to study the effect of modified creatine on rat skeletal muscle fatigue is illustrated on Fig. 1. The animals were divided into three groups in which two stimulation protocols were applied: long-term stimulation with 1 Hz frequency and tetanic stimulation with 50 Hz frequency and 5 seconds duration. The «Creatine» group was administered conventional creatine, while the «ProCreatine» group was administered a modified form of creatine (same protocol).

**Fig. 2:**
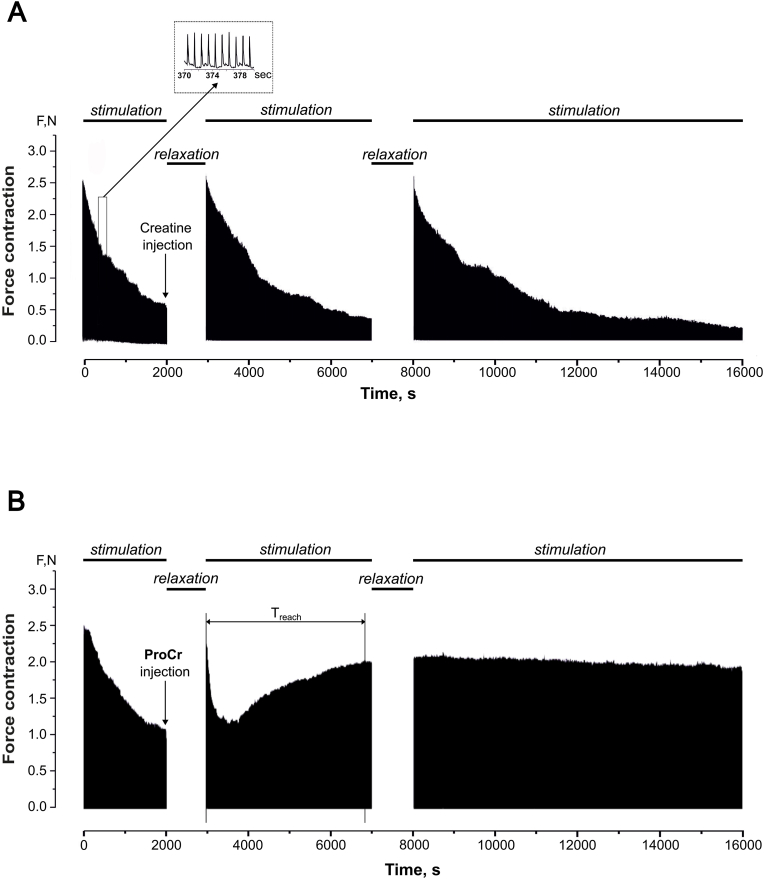
ProCr administration eliminates fatigue processes in rat gastrocnemius muscle caused by long-term stimulation at 1 Hz. (A) Stimulation duration of 2000 s reduces the maximal force activity of the muscle by 39 ± 2 % from baseline levels. The intramuscular administration of creatine monohydrate (1 mg/kg b. w., marked by arrow) did not result in a significant alteration of the muscle response amplitude after 2000 s stimulation-induced fatigue and during subsequent stimulation pools of 4000 and 8000 s duration. (B) ProCr administration (1 mg/kg b. w., marked by arrow) recovered muscle activity from stimulation-induced fatigue with the same stimulation protocol. T_reach_ indicates the period of time to reach the maximum effect of ProCr. Horizontal lines indicate the stimulation and relaxation peroids.

Figure 2 shows mechanograms of non-relaxing contractions of the gastrocnemius muscle of rats during stimulation with a frequency of 1 Hz. At 2000 s of stimulation, a decrease in the maximum strength activity of the muscle was revealed by 39 ± 2 % from the initial values. A progressive decrease in force response is a typical manifestation of fatigue processes under these stimulation conditions. The intramuscular administration of creatine monohydrate, a non-modified form of the compound, did not result in a significant alteration of the muscle response amplitude (Fig. 2A). Analysis of the simulation pool on ProCr background demonstrated the recovery of the muscle response to the initial values, which was sustained during the subsequent stimulation (Fig. 2B).

**Fig. 3:**
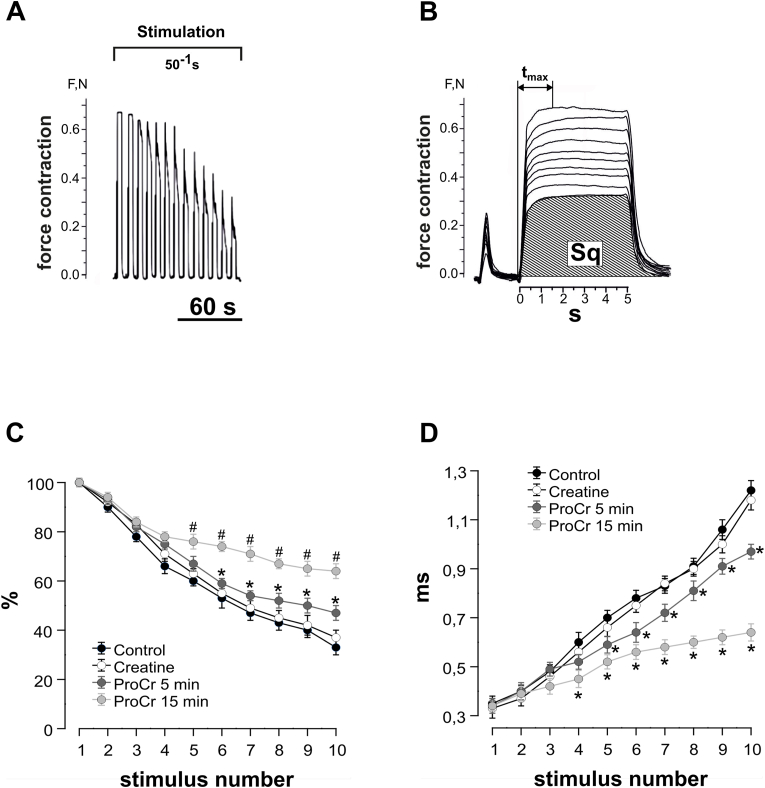
Effect of ProCr on the integral force of rat gastrocnemius muscle contractions during tetanic stimulation. (A) Original recordings of muscle responses evoked by 5 s stimulation at 50 Hz. (B) Integral force curves (only every third contraction from 30 consecutive contractions is shown): t_max_, time at which force reaches its maximum value (reaching smooth tetanus); Sq, integral muscle force calculated as the area under the force response curve. (C) Integrated muscle power as a percentage of control values (close labels), 5 min after ProCr injection (1 mg/kg b. w., dark gray labels, ∗p < 0.05) and 15 min after ProCr injection (light gray labels #p < 0.01; n = 7). Application of non-modified creatine caused no significant change in integral force (open labels, P > 0.05, n = 5). (D) Time to reach the maximum force response for control (close labels), 5 min after ProCr injection (dark gray labels, ∗p < 0.05) and 15 min after ProCr injection (light gray labels ∗p < 0.05; n = 7). In contrast, non-modified creatine had no effect in this condition (open labels, p > 0.05, n = 5).

For further analysis of mechanokinetic markers of the development of fatigue processes, 30 consecutive non-relaxation stimulations with a frequency of 50 Hz and a duration of 5 s were performed (Fig. 3A, only a fraction of the traces from the total presented). We analyzed two main parameters, which are considered to be markers of biomechanical disorders in skeletal muscles caused by factors of different nature - muscle integral force, calculated from the total area of the force curve and time to reach maximal force response (Fig. 3B) [15,16]. Markers were analyzed 5 and 15 min after drug administration.

The change in the integral force for rat gastrocnemius muscle in the control (Fig. 3C, close labels) demonstrated a significant decrease during the total period of fatigue stimulation and, at the last contraction, averaged 34 ± 2 % of the initial value. After ProCr injection, the integrated force was 43 ± 3 % (Fig. 3C, dark gray labels, p < 0.05) and 64 ± 3 % (Fig. 3C, light gray labels, p < 0.01) of the baseline value, 5 and 15 min after injection, respectively (n = 7). Application of non-modified creatine caused no significant change in integral force (Fig. 3C, open labels, p > 0.05, n = 5).

During fatigue stimulation, the time to reach the maximum force increased from 0.34 ± 0.1 s during the first contraction to 1.17 ± 0.3 s during the last one (Fig. 3D, close labels). At 5 min after ProCr administration, the similar values were 0.33 ± 0.1 s and 0.97 ± 0.2 s (Fig. 3D, dark gray labels, p < 0.05), respectively. At 15 min after ProCr administration, these values were 0.32 ± 0.1 s and 0.63 ± 0.1 s (Fig. 3D, light gray labels, p < 0.05), respectively (n = 7). In contrast, non-modified creatine had no effect in this condition (Fig. 3D, open labels, p > 0.05, n = 5).

Thus, the obtained data on the force response of the muscle against the background of muscle fatigue development indicate that administration of ProCr, unlike non-modified creatine, reduces the severity of fatigue processes.

### 3.2 Analysis of blood biochemical composition

The analysis of blood biochemical composition during the development of fatigue processes in the muscular system reflects the changes occurring in skeletal muscles and allows to evaluate the effectiveness of applied drug. We chose the following as biochemical indicators of the development of fatigue processes: creatinine, lactate, creatine phosphokinase and lactate dehydrokinase.

**Fig. 4:**
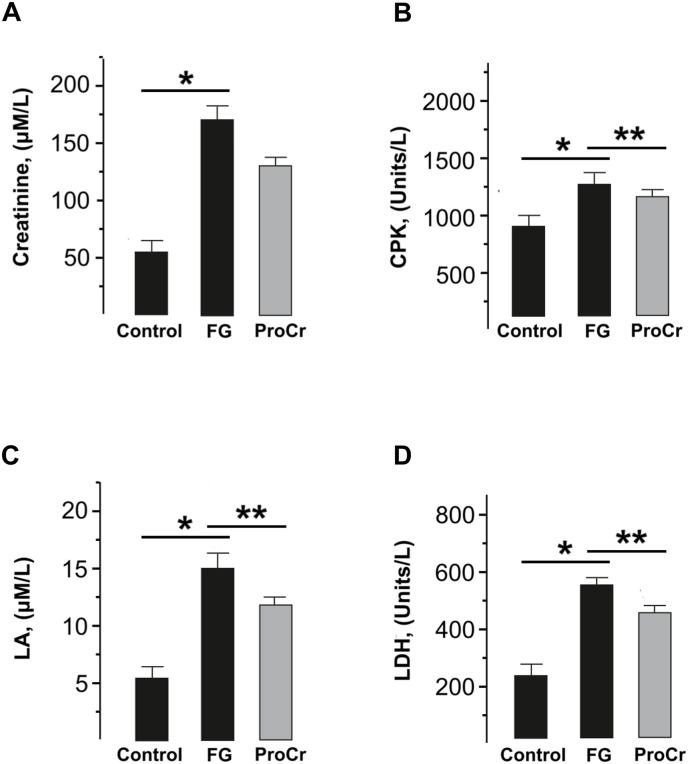
Biochemical markers of the fatigue processes development in rat blood plasma after gastrocnemius muscle contraction caused by prolonged low-frequency stimulation. (A) Creatinine; (B) CPK - creatine phosphokinase; (C) LA – lactate; (D) LDH - lactate dihydrogenase; column labels: FG – fatigue, ProCr - ProCr administration (∗p < 0.05, ∗∗p < 0.01; n = 5–6).

Changes in the level of creatinine, a product formed in muscles during the destruction of intramuscular structures during prolonged active work, make it possible to assess the level of damage to myocytes. This indicator increased from 50 ± 2 μM/L in the control to 169 ± 5 μM/L after stimulation. The protective effect of ProCr on these processes noticeably reduced this indicator to 159 ± 1 μM/l, but this decrease was not significant (Fig. 4A; p > 0.05, n = 5).

Creatine phosphokinase (CPK) is an enzyme that is found in high concentrations in skeletal muscle. The increase in the CPK blood fraction during induction of fatigue processes from 960 ± 13 U/L in control to 1380 ± 22 U/L is the result of cascade disruption of myocyte wall integrity during active prolonged contraction without relaxation. ProCr administration resulted in a significant decrease in enzyme concentration to 1289 ± 24 U/L, (Fig. 4B, p < 0.05, n = 6).

During strong muscle activity, a large amount of lactate (LA), a product of incomplete glucose oxidation, accumulates as a result of hypoxia. An increase in the level of LA in active muscle indicates that metabolic processes become anaerobic. In control condition, the LA level was 5 ± 0.4 μM/ml After the onset of fatigue, its value increased to 16 ± 1 μM/ml. ProCr injections reduced lactate levels to 12.7 ± 1 μM/ml (Fig. 4C; p < 0.01, n = 5). The level of changes in lactate dihydrogenase (LDH) an enzyme that oxidizes lactic acid, made it possible to assess the general state of muscle performance after the onset of fatigue. A significant change in the level of this enzyme from 210 ± 11 U/l in the control to 540 ± 12 U/l (Fig. 4D; p < 0.05, n = 5) after induced fatigue indicates the development of muscle dysfunction associated with an excess of fatigue-dependent derivatives. Injections of the ProCr reduced LDH concentration to 477 ± 13 units/l (p < 0.01, n = 6).

The obtained data indicate that ProCr reduces almost all biochemical parameters of muscular fatigue and is capable to compensatory activation of the muscular system under prolonged muscular exercise. It should be noted that the changes in biochemical parameters under ProCr seems to be insignificant compared to its powerful effect on contractile function and muscle performance. This discrepancy suggests our incomplete knowledge of the internal mechanisms of the fatigue process itself.

### 3.3 Effect of PrCr on smooth muscle of the rat portal vein

To study the effect further we investigated the ability of rat portal vein smooth muscles to maintain the level of isometric tension induced by field electrical stimulation before and after ProCr application.

**Fig. 5:**
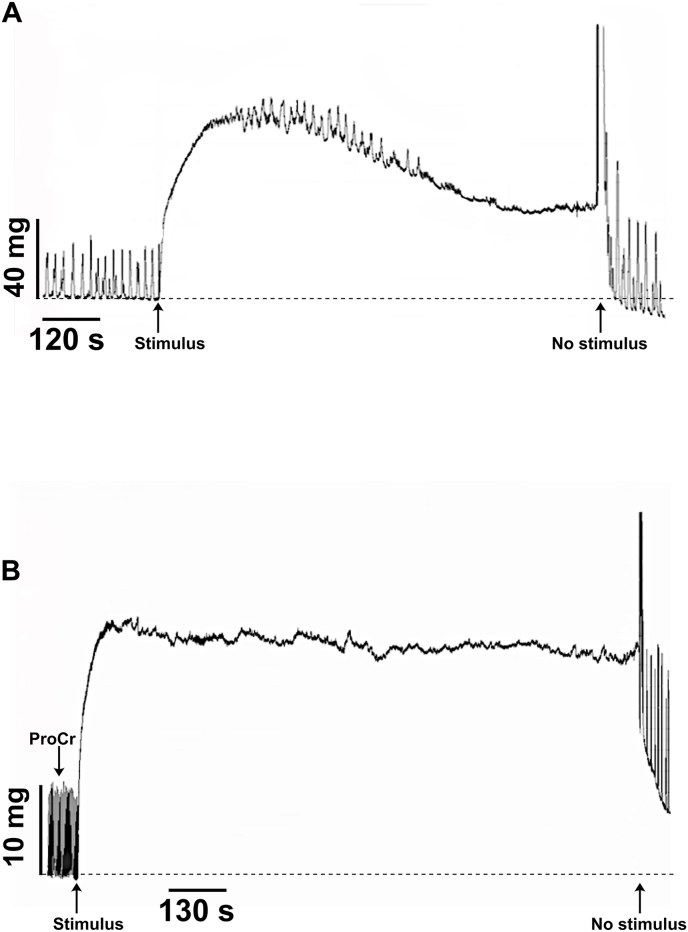
Typical original recording showing the effect of field electrical stimulation (8 Hz, 20 ms square pulses, 13 V) on spontaneously contracting rat portal vein smooth muscles in control (A) and under ProCr at 10^−4^ M (B).

Figure 5 shows the typical response of spontaneously contracting rat portal vein smooth muscle to electrical stimulation (8 Hz, 20 ms square pulses, 13 V). After reaching the maximum amplitude of contraction and the plateau, the isometric tension level starts to decrease. On average, 5 min after the beginning of stimulation, the tension value decreases to 56 % of the maximum value. This dynamics of smooth muscle isometric tension can characterise, to a certain approximation, the development of their fatigue process (Fig. 5A). Under ProCr action the level of isometric smooth muscle tension does not change significantly after a sufficiently long (more than 30 min) electrical stimulation, i.e. fatigue does not develop (Fig. 5B). Creatine monohydrate had no effect on vascular tone under the same conditions (data not shown).

## 4 Discussion

The present study examined the possibility of using a new lipophilic form of creatine ProCr as a therapeutic agent capable of reducing and correcting pathological states of muscle system resulting from fatigue processes. The main findings of this work are as follows: 1) ProCr dramatically increases the performance of the rat gastrocnemius muscle in a fatigue protocol; 2) ProCr significantly reduced the levels of creatine phosphokinase, lactate and lactate dehydrogenase as biochemical markers of muscle fatigue. 3) ProCr restored the level of rat portal vein muscle contraction while using a stimulation protocol leading to fatigue.

According to classical concepts, fatigue is a temporary decrease in the performance of a cell, organ or organism that occurs as a result of intensive work and disappears after rest. The most important feature that distinguishes muscle fatigue from muscle weakness is the ability of muscles to restore muscle strength and performance after exercise [17,18].

It is clear that all components of the muscle energy supply system are subject to energy depletion. The dynamics of depletion of energy substrates depends on the intensity and duration of mechanical work. Muscle biopsy data have convincingly shown that during short-term, tens of seconds, maximum contractions, the hydrolysis of phosphocreatine and glycogen into lactate serves as the main source of ATP regeneration [19]. If the duration of the load exceeds 1 min, then oxidative phosphorylation of intramuscular glycogen becomes the main mechanism of ATP generation. During loads lasting for hours (for example, in a marathon runner), oxidative metabolism of carbohydrates and fats provides almost all ATP production for muscle contraction [20]. At higher intensity physical loads, oxidation of carbohydrates predominates, especially from muscle glycogen, whereas at lower intensity, oxidation of fats is more important [21].

Another important intermediate product of metabolism is lactic acid. Increasing oxygen deficiency leads to an exponential increase in the concentration of lactic acid, which, as a result of dissociation, leads to the appearance of excess hydrogen ions (H^+^). i.e. to acidosis [22]. A decrease in pH from 7.1 to 7.3 (under physiological conditions) to 6.6–6.4 (during intense muscle load) negatively affects the activity of various enzymes, including phosphofructokinase, which is involved in the process of glycolysis and directly affects the rate of ATP formation. A decrease in pH to 6.4 leads to a rapid drop in ATP levels and, as a consequence, to the development of muscle fatigue [19].

It has been previously hypothesized that muscle fatigue is caused by changes in ion concentrations in muscle. This assumption was based on data demonstrating that after repeated and prolonged exercise, the velocity of action potential (AP) propagation changes, as well as the ratio of ions in muscle fibers and interstitial fluid. Thus, after prolonged exercise, the content of potassium ions (K^+^) in the interstitial fluid increases, which leads to a decrease in AP amplitude [23]. Allen et al. described an important mechanism for changes in intracellular signaling processes, in which a decrease in AP amplitude during the development of fatigue inhibits the release of intracellular calcium from the sarcoplasmic reticulum [24]. It has also been shown that substances that directly or indirectly activate the ATP-dependent sodium-potassium pump maintain calcium release, which can delay the onset of fatigue and reduce the severity of its manifestations [25].

The problem of smooth muscle (SM) fatigue is poorly studied. Obviously, this is due to the generally accepted idea that in muscles that are constantly in the state of a certain level of tonic tension, it is either not expressed at all or develops almost imperceptible.

Nevertheless, Pagala et al. demonstrated that physiological fatigue of the bladder SM was recorded already after 60 s of electrical stimulation [26]. In this case, contractile fatigue is manifested in both longitudinal and transverse bands of the bladder SM. Increasing the stimulation frequency from 5 to 30 Hz increased the degree and rate of fatigue development. They concluded that fatigue of the bladder SM smooth muscles may be due to several mechanisms: changes in the level of polarization of smooth muscle membranes, a decrease in the release of a mediator from nerve endings, or other as yet unexplored processes.

Importantly, vascular smooth muscle (VSM) is highly dependent on glycolysis and is characterized by a significant rate of glycolysis producing lactate even under oxygenated conditions. It has been suggested that this phenomenon does not represent a defect in vascular metabolism but is related to Na^+^/K^+^ transport, whereas oxidative metabolism is more related to the development of isometric smooth muscle contraction [27]. Interestingly, glycolysis can also provide l-lactate levels, which serves as an energy substrate that increases mitochondrial reserve capacity, and mediate the regulation of gene proliferation, migration, and transcription through the expression of mTOR kinase and adenosine monophosphate-activated protein kinase (AMPK), components of the vascular injury signaling pathway [28].

Although energy consumption is lower in SM than in striated muscles (such as heart and skeletal muscle), creatine kinase (CK) is nevertheless present at relatively high levels. An important CK isoform in smooth muscle from an energetic aspect is mitochondrial CK (Mi-CK). Multiple CK isoforms may be located within the cell, performing specific functions associated with other localised proteins and metabolites [29].

Thus, the current understanding of the fatigue mechanisms in the muscular system does not allow us to conclude that there is a unified mechanism underlying this process. This is even more true for smooth muscles. However, it is clear that creatine plays a key role in the supply and transport of cellular energy, and there is no doubt that the role of energy metabolism is extremely important for muscle performance. In this context, continuous transport of creatine into the cell may be an important factor in ensuring effective muscle performance over a long period of time. It is known that exogenous creatine supplementation can increase the level of the muscle glucose transporter GLUT4, muscle glycogen and muscle creatine and thereby promote both the flow of glucose and its subsequent uptake into cellular metabolism and an increase in ATP levels via the CK pathway [30]. In addition to the extensive evidence supporting the ergogenic benefits of creatine, recent data suggest a much broader use of creatine in various myopathies, neurodegenerative diseases and other pathologies. Furthermore, creatine has been found to exhibit non-ergogenic properties, acting as a direct and indirect antioxidant, causing anti-inflammatory effects [7].

It is of interest to note that the effective, and indeed somewhat paradoxical, effect of ProCr on the contractility and performance of skeletal and smooth muscles during the development of fatigue, as demonstrated in our experiments, does not correlate with the biochemical parameters of this process. The biochemical markers of fatigue exhibited modest alterations upon ProCr administration with the exception of free lactate, which demonstrated a notable reduction with both creatine and ProCr. Nevertheless, the distinction in contractile effects is substantial, prompting reconsideration of the hypothesis that the energetic component of fatigue is the primary mechanism underlying its development. The data obtained may indicate that ionic mechanisms of fatigue are predominant, with changes in the excitability of cellular muscle membranes.

The elevation in ATP levels resulting from purine nucleotide rephosphorylation may be associated with blockade of ATP-sensitive potassium channels and augmented Ca^2+^ influx during repolarisation, which enhances the force of contraction and duration of repolarisation [31].

This insight into the mechanisms of fatigue development requires experimental confirmation, which will be the objective of our further studies. Moreover, the alkalinity of creatine entering by diffusion rather than through a transporter may enhance muscular performance by maintaining the required pH level.

In conclusion, the results demonstrate that modified creatine has the ability to easily penetrate cell membranes without transporter involvement. This facilitates an increase in the intracellular creatine pool, which in turn inhibits the processes leading to muscle fatigue.

## CRediT authorship contribution statement

**Anatoly Soloviev:** Writing – review & editing, Supervision, Conceptualization. **Vadym Kozlovsky:** Methodology, Conceptualization. **Dmytro Nozdrenko:** Investigation, Formal analysis. **Vadym Sydorenko:** Writing – original draft. **Igor Monchak:** Investigation, Formal analysis. **Natalia Vdovenko:** Investigation, Formal analysis. **Olena Maidaniuk:** Investigation, Formal analysis. **Volodymyr Fetyukhin:** Investigation.

## Funding

The authors declare that no funds, grants, or other support were received during the preparation of this manuscript.

## Declaration of competing interest

The authors: Kozlovskyi V., Fetuchin V. own shares of 20% in a company that will potentially produce procreatine-based products. All authors did not receive any financial rewards for their research and have no prejudice against any creatine-based products: the research interests were defined as scientific exclusively.

## Data Availability

All data generated or analyzed during this study are included in this article. Raw files and additional information may be available by the corresponding author upon reasonable request.
